# Prediction model for cognitive impairment in maintenance hemodialysis patients

**DOI:** 10.1186/s12883-023-03407-z

**Published:** 2023-10-12

**Authors:** Ding Chen, Chang Xiao, Wangyan Xiao, Linjing Lou, Zhuo Gao, Xinlun Li

**Affiliations:** Department of Nephrology, Air Force Medical Center, PLA, No.30, Fucheng Road, Haidian District, Beijing, 100037 China

**Keywords:** Prediction model, Cognitive impairment, End-stage renal disease, Maintenance hemodialysis

## Abstract

**Purpose:**

To explore the risk factors for cognitive impairment in patients undergoing maintenance hemodialysis (MHD) and construct a predictive model for cognitive impairment.

**Methods:**

A total of 146 patients with end-stage renal disease (ESRD) undergoing MHD were recruited at our hospital between December 2021 and April 2022. Cognitive function was assessed using the Montreal Cognitive Assessment (MoCA), and scores of < 26 were considered indicative of cognitive impairment. Risk factors were identified using a multivariate logistic regression model, and a receiver operating characteristic curve was applied to construct the prediction model. Cognitive impairment risk was categorized using a multifactorial prediction model based on the weight of evidence.

**Results:**

46 patients with cognitive impairment were identified, with a prevalence of 31.5% in ESRD patients undergoing MHD. Multivariate logistic regression analyses indicated that the following factors were associated with an increased risk of cognitive impairment in patients undergoing MHD: aged 55.0–64.0 years (OR:6.24; 95%CI:1.81–21.48; *P* = 0.001), aged 65.0–74.0 years (OR:16.10; 95%CI:4.03–64.37; *P* < 0.001), aged ≥ 75.0 years (OR:90.22; 95%CI:16.86-482.86; *P* < 0.001), duration of dialysis ≥ 5 years (OR:3.99; 95%CI:1.58–10.04; *P* = 0.003), and current smoker (OR:4.61; 95%CI:1.46–14.57; *P* = 0.009). The predictive value of the constructed model based on the aforementioned factors for cognitive impairment was 84% (95%CI,77-91%). The prevalence of cognitive impairment for patients at low, moderately low, moderately high, and high risk was 0% (95%CI:0-17%), 10% (95%CI:3-22%), 32% (95%CI:16-52%), and 65% (95%CI:50-78%), respectively.

**Conclusions:**

This study constructed a multifactorial prediction model with a high predictive value for cognitive impairment in patients with ESRD undergoing MHD.

## Introduction

Chronic kidney disease (CKD) is present in one-eighth of the adult population, and this proportion rises to one-third for individuals aged over 65 years [1, 2]. CKD has been significantly associated with an elevated risk of cardiovascular and cerebrovascular diseases, end-stage renal disease (ESRD), and early mortality [3–5]. CKD is an independent risk factor for dementia and cognitive impairment [6]. Studies have found that changes in cognitive impairment begins at the early stage of CKD, and that there is a strong relationship between decreased kidney function and impaired cognition [7, 8]. The prevalence of cognitive impairment in patients with CKD, and ESRD especially, can reach 80% [9, 10]. Informed decisions and compliance with dialysis activities could be affected by cognitive impairment, which is considered an independent risk factor for mortality in ESRD patients undergoing hemodialysis [11–13]. Thus, identifying cognitive impairment is particularly important for early prevention and slowing the progression of cognitive decline in patients undergoing maintenance hemodialysis (MHD).

The potential causes of cognitive impairment in patients are multifactorial, including cerebrovascular lesions [14], hypotension [15], serological markers [16], social history [17], and frequency of hemodialysis [18]. Moreover, several studies have illustrated the impact factors for cognitive impairment in patients undergoing hemodialysis and identified predictive factors, including older age, duration of education, undergoing hemodialysis, hemoglobin level, depression, and smoking [19–21]. Identifying MHD patients at high risk for cognitive impairment is particularly important; however, no prediction model has been constructed to predict cognitive impairment in MHD patients. Therefore, the current study was performed to identify potential predictive factors for cognitive impairment in patients undergoing MHD, and a prediction model was constructed based on these identified predictive factors. Furthermore, a categorical risk-scoring system was created to evaluate the risk of cognitive impairment in patients undergoing MHD in a specific risk category.

## Materials and methods

### Population

ESRD patients undergoing MHD in our hospital between December 2021 and April 2022 were collected retrospectively. Patients were included if they met the following criteria: (1) age ≥ 18.0 years; (2) regular hemodialysis ≥ 3.0 months; and (3) ability to complete the cognitive function questionnaire. The exclusion criteria were as follows: (1) history of invasive craniocerebral surgery; (2) mental illness, including Alzheimer’s disease or schizophrenia; (3) history of traumatic brain injury; (4) neurological diseases, such as ischemic or hemorrhagic stroke; (5) long-term use of antiepileptic or antipsychotic drugs; (6) chronic alcoholism; and (7) history of acute cardiovascular and cerebrovascular disease, trauma, surgery, acute infection, or other stresses experienced within the past one month. The institutional review board of the hospital approved this study and written informed consent was obtained from the patients after explaining the purpose of the study.

### Data collection and variable definition

All data was collected from self-questionnaires, interviews, and medical records, and a total of 23 factors were considered, including sex (male, female), age (< 55.0, 55.0–64.0, 65.0–74.0, ≥ 75.0 years), body mass index (BMI: < 20.0, 20.0–25.0, ≥ 25.0 kg/m^2^), weight gain proportion (0–5.0%, ≥ 5.0%), duration of dialysis (0–5.0, ≥ 5.0 years), hypertension (yes, no), diabetes mellitus (DM: yes, no), cardiovascular disease (CVD: yes, no), smoking (yes, no), insomnia (yes, no), hemoglobin (low: < 110 g/L; normal:110–130 g/L), albumin (low: < 35.0 g/L; normal:35.0–51.0 g/L), blood urea nitrogen (BUN, high: > 7.0 mmol/L; normal:3.2–7.1 mmol/L), uric acid (high: ≥ 420 umol/L for male and ≥ 360 umol/L for female; normal:180–420 umol/L for male and 120–360 umol/L for female), triglyceride (TG, high: > 1.21 mmol/L; normal:0.20–1.21 mmol/L), total cholesterol (TC, low: < 2.86 mmol/L; normal:2.86–5.98 mmol/L), high density lipoprotein (HDL, low: < 1.08 mmol/L; normal:1.08–1.91 mmol/L), low density lipoprotein (LDL, high: > 3.37 mmol/L; normal: ≤ 3.37 mmol/L), high-sensitive C-reaction protein (hs-CRP, high: > 0.88; normal:0.68–0.88), parathyroid hormone (PTH, high: > 300 pg/mL; normal:150–300 pg/mL), vitamin D (high: > 150.0 nmol/L; normal:50.0-150.0 nmol/L; low: < 50.0 nmol/L), spKt/V (normal: ≥ 1.2; low: < 1.2), alkaline phosphatase (ALP, high: > 120 U/L for male and > 130 U/L for female; normal:45–120 U/L for male and 50–130 U/L for female; low: < 45 U/L for male and < 50 U/L for female), and ferritin (high: > 500.0 ug/L; normal:200–500.0 ug/L; low: < 200 ug/L). Data collection was performed by a clinician and a nurse after training.

### Cognitive impairment

The Montreal Cognitive Assessment (MoCA) was applied to assess cognitive function. The MoCA is a 30-point screening tool which takes patients approximately 10 min to complete [22–24]. Multiple domains are identified in the MoCA, including memory, visuospatial abilities, executive function, language, attention, concentration, working memory, and orientation to time and place. This cognitive function assessment was administered in a quiet room by medical staff who had been trained by psychologists. MoCA scores are adjusted for educational level, and an additional score should be added to the total score for patients with ≤ 12 years of formal education. The MoCA score system ranges from to 0–31, and the presence of cognitive impairment is determined at scores < 26 [22].

### Statistical analysis

The characteristics of patients with and without cognitive impairment were collected as categorical and continuous data, presented as frequencies (proportions) and medians (interquartile ranges), respectively. The differences between groups was assessed using the Kruskal-Wallis method for continuous data, while the Chi-square or Fisher’s test was applied to assess differences in categorical data. Potential predictive factors were identified using univariate logistic regression analysis, and the factors were subjected to multivariate analyses using α = 0.05 and β = 0.10. Multivariate logistic regression was conducted to explore the predictive factors for cognitive impairment after adjusting for potential confounding factors. The weight of evidence was obtained from the regression coefficients for specific factors that were used to construct a categorical risk-scoring system. The numbers rounded to the nearest unit was applied as scoring values. Subsequently, a prediction model was constructed by combining predictive factors, and the predictive value was assessed using the receiver operating characteristic (ROC) curve with the area under the curve (AUC). Statistical significance was defined as a two-tailed *P* < 0.05. All statistical analyses were performed using SPSS Version 18 for Windows (SPSS Inc., Chicago, IL, USA).

## Results

### Baseline characteristics of included patients

Of the 146 included patients (96 male and 50 female), 46 patients presented with cognitive impairment (a prevalence of 31.5%). The median age of the patients was 56.0 years. The baseline characteristics of the participants are shown in Table 1. There were significant differences between patients with and without cognitive impairment for the following factors: age (*P* < 0.001), cardiovascular disease (*P* = 0.006), low vitamin D level (*P* = 0.036), and ferritin (*P* = 0.002). No significant differences were found between groups for: sex, BMI, weight gain, duration of dialysis, hypertension, DM, smoking, insomnia, hemoglobin, albumin, BUN, uric acid, TG, TC, HDL, LDL, hs-CRP, PTH, spKt/V, and ALP.

**Table 1:** Baseline characteristics of included patients

Variables	Overall (n = 146)	Cognitive impairment		OR	*P* value
		No (n = 100)	Yes (n = 46)		
Sex (%)					0.924
Male	96 (65.75)	65 (65.00)	31 (67.39)	1	
Female	50 (34.25)	35 (35.00)	15 (32.61)	0.90 (0.42–1.88)	
Age	56.00 (46.00,67.00)	50.00 (41.00,60.25)	67.50 (59.00,82.50)	1.09 (1.05–1.12)	< 0.001
Age group (years)					< 0.001
< 55.0	66 (45.21)	57 (57.00)	9 (19.57)	1	
55.0–64.0	39 (26.71)	27 (27.00)	12 (26.09)	2.77 (1.04–7.68)	
65.0–74.0	23 (15.75)	12 (12.00)	11 (23.91)	5.63 (1.91–17.3)	
≥ 75.0	18 (12.33)	4 (4.00)	14 (30.43)	20.4 (5.87–88.9)	
BMI (%)					0.147
Normal	74 (50.68)	46 (46.00)	28 (60.87)	1	
Underweight	40 (27.40)	28 (28.00)	12 (26.09)	0.71 (0.30–1.61)	
Overweight	32 (21.92)	26 (26.00)	6 (13.04)	0.39 (0.13–1.02)	
Weight gain (%)					0.742
0–5	94 (64.38)	63 (63.00)	31 (67.39)	1	
≥ 5	52 (35.62)	37 (37.00)	15 (32.61)	0.83 (0.39–1.72)	
Duration of dialysis (years)					0.092
0–5	80 (54.79)	60 (60.00)	20 (43.48)	1	
≥ 5	66 (45.21)	40 (40.00)	26 (56.52)	1.94 (0.96–3.98)	
Hypertension					0.315
No	1 (0.68)	0 (0.00)	1 (2.17)	1	
Yes	145 (99.32)	100 (100.00)	45 (97.83)	.(.-.)	
Diabetes mellitus					0.372
No	104 (71.23)	74 (74.00)	30 (65.22)	1	
Yes	42 (28.77)	26 (26.00)	16 (34.78)	1.52 (0.70–3.23)	
Cardiovascular disease					0.006
No	80 (54.79)	63 (63.00)	17 (36.96)	1	
Yes	66 (45.21)	37 (37.00)	29 (63.04)	2.87 (1.40–6.05)	
Smoking					0.730
No	109 (74.66)	76 (76.00)	33 (71.74)	1	
Yes	37 (25.34)	24 (24.00)	13 (28.26)	1.25 (0.55–2.74)	
Insomnia					0.932
No	107 (73.29)	74 (74.00)	33 (71.74)	1	
Yes	39 (26.71)	26 (26.00)	13 (28.26)	1.12 (0.50–2.45)	
Hemoglobin (g/L)					0.491
Normal	60 (41.10)	43 (43.00)	17 (36.96)	1	
Low	86 (38.90)	57 (57.00)	29 (63.04)	1.29 (0.63–2.64)	
Albumin (g/L)					1.000
Normal	140 (95.89)	96 (96.00)	44 (95.65)	1	
Low	6 (4.11)	4 (4.00)	2 (4.35)	1.13 (0.14–6.37)	
BUN (mmol/L)					0.797
Normal	19 (13.01)	14 (14.00)	5 (10.87)	1	
Elevated	127 (86.99)	86 (86.00)	41 (89.13)	1.31 (0.46–4.38)	
Uric acid (umol/L)					0.285
Normal	62 (42.47)	39 (39.00)	23 (50.00)	1	
Elevated	84 (57.53)	61 (61.00)	23 (50.00)	0.64 (0.31–1.30)	
TG (mmol/L)					0.932
Normal	69 (47.26)	48 (48.00)	21 (45.65)	1	
Elevated	77 (52.74)	52 (52.00)	25 (54.35)	1.10 (0.54–2.23)	
TC (mmol/L)					1.000
Normal	121 (82.88)	83 (83.00)	38(82.61)	1	
Low	25 (17.12)	17 (17.00)	8 (17.39)	1.04 (0.39–2.57)	
HDL (mmol/L)					0.660
Normal	40 (27.40)	29 (29.00)	11 (23.91)	1	
Low	106 (72.60)	71 (71.00)	35 (76.09)	1.29 (0.59–2.99)	
LDL (mmol/L)					1.000
Normal	141 (96.58)	96 (96.00)	45 (97.83)	1	
Elevated	5 (3.42)	4 (4.00)	1 (2.17)	0.59 (0.02–4.40)	
Hs-CRP					0.086
Normal	110 (75.34)	80 (80.00)	30 (65.22)	1	
Elevated	36 (24.66)	20 (20.00)	16 (34.78)	2.12 (0.96–4.67)	
PTH (pg/mL)					0.919
Normal	115 (78.77)	79 (79.00)	36 (78.26)	1	
Elevated	31 (21.23)	21 (21.00)	10 (21.74)	1.04 (0.45–2.44)	
Vitamin D (nmol/L)					0.036
Normal	39 (26.71)	32 (32.00)	7 (15.22)	1	
Low	106 (72.60)	67 (67.00)	39 (84.78)	2.66 (1.07–6.60)	
Elevated	1 (0.68)	1 (1.00)	0 (0.00)	1.44 (0.05–39.07)	
spKt/V					0.055
Normal	105 (71.92)	67 (67.00)	38 (82.61)	1	
Low	41 (28.08)	33 (33.00)	8 (17.39)	0.43 (0.18–1.02)	
ALP (U/L)					0.614
Normal	103 (70.55)	72 (72.00)	31 (67.39)	1	
Low	9 (6.16)	7 (7.00)	2 (4.35)	0.70 (0.09–3.19)	
Elevated	34 (23.29)	21 (21.00)	13 (28.26)	1.44 (0.62–3.23)	
Ferritin (ug/L)					0.002
Normal	49 (33.56)	25 (25.00)	24 (52.17)	1	
Low	73 (50.00)	54 (54.00)	19 (41.30)	0.37 (0.17–0.79)	
Elevated	24 (16.44)	21 (21.00)	3 (6.52)	0.15 (0.04–0.56)	

### Multivariate analysis

**Fig. 1:**
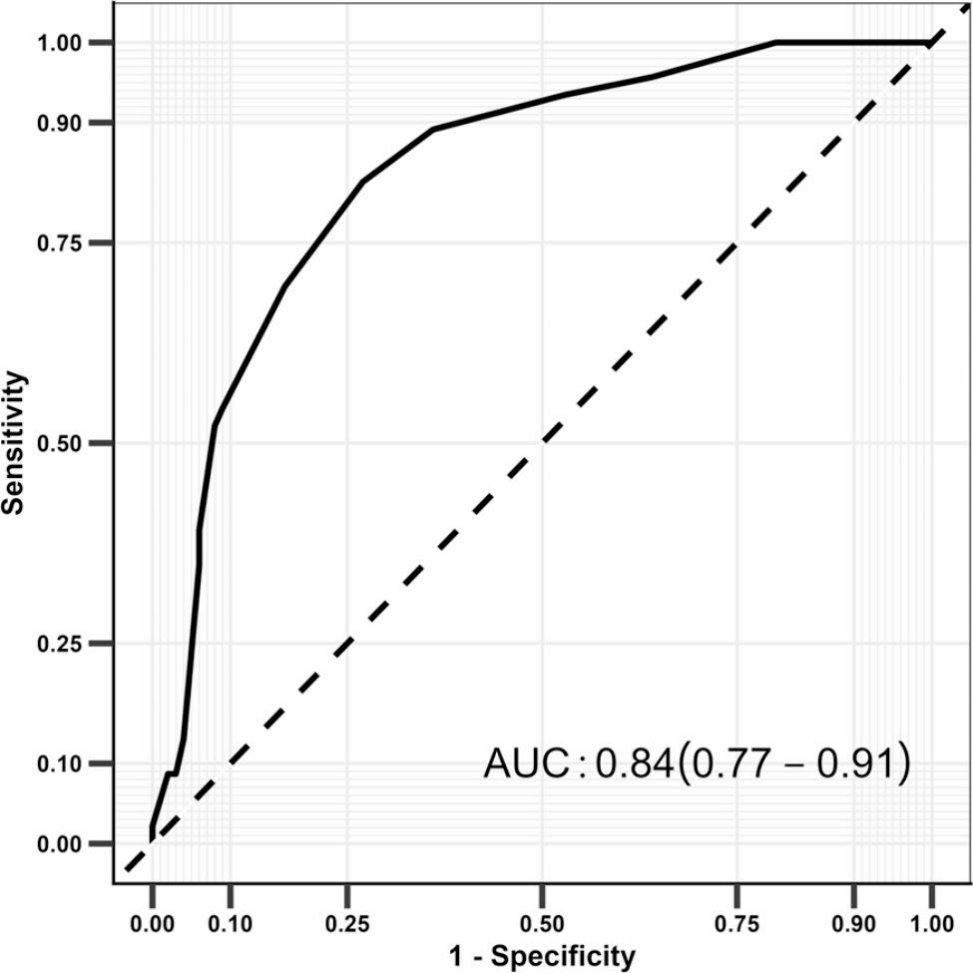
Receiver operating characteristic curve for the risk of cognitive impairment in ESRD patients undergoing MHD, including the 3-component risk factor model

Multivariate stepwise logistic regression was performed after adjusting for potential confounding factors, and the results are shown in Table 2. The following factors were associated with an increased risk of cognitive impairment in ESRD patients undergoing MHD: aged 55.0–64.0 years (OR:6.24; *P* = 0.001), aged 65.0–74.0 years (OR:16.10; *P* < 0.001), aged ≥ 75.0 years (OR:90.22; *P* < 0.001), duration of dialysis ≥ 5 years (OR:3.99; *P* = 0.003), and current smoker (OR:4.61; *P* = 0.009). Subsequently, the above factors were combined to construct the prediction model, and the ROC curve was drawn, with an AUC of 84% (95%CI: 77-91%) (Fig. 1).

**Table 2:** Risk factors in the prediction model for cognitive impairment using multivariate logistic regression

Variables		β	OR	95%CI		χ^2^	*P* value
				Lower	Upper		
Constant		-3.60				28.636	< 0.001
Age (years)	< 55.0	Reference					
	55.0–64.0	1.83	6.24	1.81	21.48	8.415	0.004
	65.0–74.0	2.78	16.10	4.03	64.37	15.447	< 0.001
	≥ 75.0	4.50	90.22	16.86	482.86	27.672	< 0.001
Duration of dialysis (years)	0–5	Reference					
	≥ 5	1.38	3.99	1.58	10.04	8.616	0.003
Smoking	No	Reference					
	Yes	1.53	4.61	1.46	14.57	6.757	0.009

### Risk scoring system

**Fig. 2:**
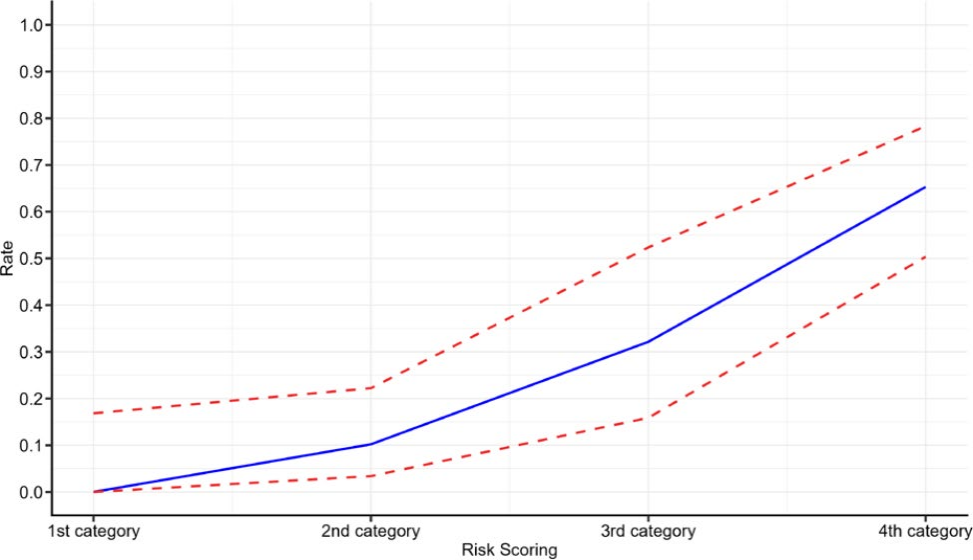
Probability of developing cognitive impairment for patients at specific-risk category

The risk scoring system was established on the basis of a multivariate logistic regression model, and cognitive impairment risk according to four categories is shown in Fig. 2. The number of patients in 1st, 2nd, 3rd, and 4th categories was 20, 49, 28, and 49, respectively, and the prevalence of cognitive impairment was 0% (95%CI:0-17%), 10% (95%CI:3-22%), 32% (95%CI:16-52%), and 65% (95%CI:50-78%), respectively.

## Discussion

The current study is the first to construct a multifactorial predictive model for the risk of cognitive impairment in ESRD patients undergoing MHD which can be used as a predictive tool for identifying patients at high risk of cognitive impairment. Consequently, preventive strategies could be used to adjust modifiable risk factors and improve prognosis. A total of 146 patients with ESRD undergoing MHD were identified, and the prevalence of cognitive impairment was 31.5%. The prediction model was constructed based on age, dialysis duration, and smoking status, and the predictive value of the constructed model was high.

The prevalence of cognitive impairment found in this study is consistent with prior studies, which observed cognitive impairment in 30-80% of patients undergoing hemodialysis [9, 25, 26]. In our study, patients with a history of mental illness or neurological disease were excluded, which could partly explain the comparatively lower incidence of cognitive impairment in patients undergoing MHD.

Studies have demonstrated that CKD is associated with an increased risk of cerebrovascular disease, which can in turn induce cognitive impairment [27]. Moreover, cerebral perfusion pressure is related to a decrease in blood flow velocity due to a sharp decrease in blood volume in blood vessels caused by dialysis dehydration, which can cause a decline in cognitive function [28]. Furthermore, repeated cyclic stress of hemodialysis can lead to ischemic brain injury owing to the repeated decrease in cerebral blood flow during hemodialysis, which could induce an accelerated decline in cognitive function [29].

Several studies have identified risk factors for cognitive impairment in patients undergoing hemodialysis [19–21]. Drew et al. studied 314 older patients and identified age as the only significant risk factor affecting the rate of executive function decline [19]. Odagiri et al. studied 154 hemodialysis patients and 852 participants from the general population, and found that age and hemodialysis were significantly associated with an increased risk of cognitive impairment [20]. Karakizlis et al. prospectively collected 408 patients and found that sex, hemoglobin level, depression, and smoking could affect the progression of cognitive impairment in patients undergoing hemodialysis [21]. Our study identified risk factors for cognitive impairment, including age, duration of dialysis, and smoking status. Several considerations could explain these results: (1) Older patients have a higher prevalence of cardiovascular risk factors, which are, in turn, stronger risk factors for cognitive impairment [21]. Moreover, aging is related to changes in the brain which could affect cognitive function, including general atrophy (particularly in the hippocampus), imbalance of amyloid-β production and degradation, inflammatory response, and frailty of neurons, [30]. Furthermore, the neuronal metabolism, function and survival could affected by aging, which contributed an important role on the progression of cognitive declie and neurodegenerative diseases [31, 32]; (2) The duration of dialysis is significantly related to the severity of ESRD and kidney function, and cognitive impairment may occur prior to kidney failure [7]. (3) Cigarette smoking could accelerate brain aging, and metals in cigarette smoke could accumulate in tissues and fluids, causing heavy metal toxicity and promoting cognitive impairment [33–36].

Our study constructed a prediction model for cognitive impairment in patients with ESRD undergoing MHD, and the three risk factors were combined. The AUC of the constructed model was 84% (95%CI:77-91%), which suggests a high predictive value for cognitive impairment. Moreover, a risk scoring system was established, and the prevalence of cognitive impairment was estimated as 0%, 10%, 32%, and 65%. Thus, patients at high risk for cognitive impairment should be carefully monitored, and prevention and early treatment strategies should be applied to prevent the risk of cognitive impairment and improve the prognosis.

Several limitations of this study should be acknowledged. First, it was designed using a retrospective cohort, and the results could be affected by various confounding factors. Second, the severity of ESRD was not addressed, which might have affected the prevalence of cognitive impairment. Third, cognitive impairment contains various domains, which should be further analyzed to determine the potential impacts of MHD. Fourth, the analysis was based on a small sample size, and the constructed model lacked external validation.

## Conclusions

Our study constructed a predictive model for cognitive impairment in ESRD patients undergoing MHD. A total of three factors were identified and subjected to the prediction model, including age, duration of dialysis, and smoking status, and the predictive value of the constructed model was high. Thus, the current prediction model should be applied in clinical practice and further large-scale prospective cohort studies should be performed to validate the predictive value of the constructed model.

## Data Availability

The datasets generated during and/or analysed during the current study are available from the corresponding author on reasonable request.
